# Advances in the Endoscopic Management of Obesity

**DOI:** 10.1155/2015/757821

**Published:** 2015-05-28

**Authors:** Jason Behary, Vivek Kumbhari

**Affiliations:** ^1^Department of Medicine and Division of Gastroenterology and Hepatology, St George Hospital, Kogarah, Sydney, NSW 2217, Australia; ^2^Department of Medicine and Division of Gastroenterology and Hepatology, Johns Hopkins Medical Institutions, Baltimore, MD 21287, USA

## Abstract

Obesity has become a worldwide epidemic with significant impact on quality of life, morbidity, and mortality rates. Over the past two decades, bariatric surgery has established itself as the most effective and durable treatment for patients with obesity and its associated comorbidities. However, despite the use of minimally invasive techniques, bariatric surgery is associated with complications in approximately 15% of patients, has a substantial cost, and is used by only 1% of patients who are eligible. Therefore, there is a need for effective minimally invasive therapies, which will be utilized by the large proportion of obese patients who are in desperate need of treatment but are not receiving any. Endoscopic approaches to the management of obesity have been developed, with the aim of delivering more effective, durable, and safer methods of weight reduction. In this paper, we review currently available and future endoscopic therapies that will likely join the armamentarium used in the management of obesity.

## 1. Background

Obesity is a complex chronic disease which results in an increased morbidity and mortality [1–3]. Its growing prevalence and associated comorbidities, particularly the development of type 2 diabetes mellitus, have a significant impact on quality of life and impose a large economic burden on healthcare systems [4]. The treatment of obesity remains a difficult clinical problem and, despite initial weight loss, maintaining a healthy weight is not possible in the majority of patients [5, 6]. Currently, accepted therapies for the management of obesity include dietary modification, physical exercise, pharmacological treatment, surgical therapy, and, more recently, endoscopic treatment.

Dietary and lifestyle modification often fail to achieve desired weight loss outcomes [7]. Pharmacological therapies are often associated with contraindications and low rates of compliance [8, 9]. In the United States, the National Institute of Health recommends weight loss surgery as an option for carefully selected individuals with a body mass index (BMI) of ≥40 kg/m^2^ or those with a BMI of ≥35 kg/m^2^ with significant comorbidities, who have failed diet, exercise, and drug therapy [10].

Several surgical approaches (collectively referred to as “bariatric surgery”) have been successfully used to treat obesity. In a systematic review and meta-analysis of randomised controlled trials, there were greater weight loss (mean difference loss of 26 kg; 95% CI 21–31) and higher remission rates for type 2 diabetes mellitus (relative risk 22.1; CI 3.2–154.3) in the bariatric surgery group compared to lifestyle modification alone [7, 11, 12]. Newer laparoscopic bariatric surgical techniques provide multiple advantages over older, open surgical methods. These include lower postoperative adverse events, earlier ambulation after surgery, and reduced length of hospital stay [13, 14]. Notably, bariatric surgery is associated with a significant improvement in comorbidities and has been proven to reduce mortality [15, 16].

Despite the clear benefits of bariatric surgery, there are some pitfalls. Importantly, bariatric surgery is associated with significant morbidity and substantial costs [17, 18]. In addition, bariatric surgery is not available to patients with a BMI <35 kg/m^2^ even if clinically significant comorbidities (metabolic, psychological, etc.) exist. Additionally, only a minute proportion of patients who qualify and would conceivably benefit from bariatric surgery ever undertake it [16]. Finally, the durability of bariatric surgery has recently been questioned with weight regain being not uncommon [19–21].

Current research is focused on the development of alternative methods of obesity treatment that are less invasive, more cost-effective, and associated with a lower operative risk. Such methods should also be efficacious, durable, repeatable, reversible, and safe. Endoluminal interventions performed entirely through the gastrointestinal (GI) tract, using flexible endoscopy, offer the potential for ambulatory weight loss procedures that may be safer and more cost-effective than current laparoscopic approaches.

Endoscopy has a well-established role in the preoperative evaluation of patients undergoing bariatric surgery and in the assessment and management of surgical complications [22]. In addition, endoscopic procedures have been used as a “bridge to surgery” in order to reduce obesity related operative risks. However, endoscopic therapies as a primary treatment modality for obesity have only more recently been explored. Endoscopic modalities in the treatment of obesity can be categorised into the following: space-occupying devices, gastric restrictive methods, malabsorptive endoscopic procedures, regulating gastric emptying, and other therapies. Of these methods, the most commonly employed are space-occupying devices. The review outlines and discusses currently available and future endoscopic therapies as a primary treatment modality for obesity.

## 2. Space-Occupying Devices

### 2.1. Intragastric Balloons

Intragastric balloons (IGB) have been used in the treatment of obesity for the last 20 years with a large body of evidence supporting its short-term efficacy and safety.

Initial balloons constructed from gum and latex were not resistant to gastric acid and deflated quickly [23]. The Garren-Edwards gastric bubble (American-Edwards Laboratories, USA) was an air-filled polyurethane balloon that was approved by the US Food and Drug Administration in 1985. The balloon was widely used for several years but was later withdrawn from use when several studies demonstrated its lack of superiority to diet and behavioural therapy [24, 25]. These balloons were also associated with a large number of serious complications including mucosal erosion (26%), gastric ulcer (14%), and small bowel obstruction (2%) [26].

**Figure 1:**
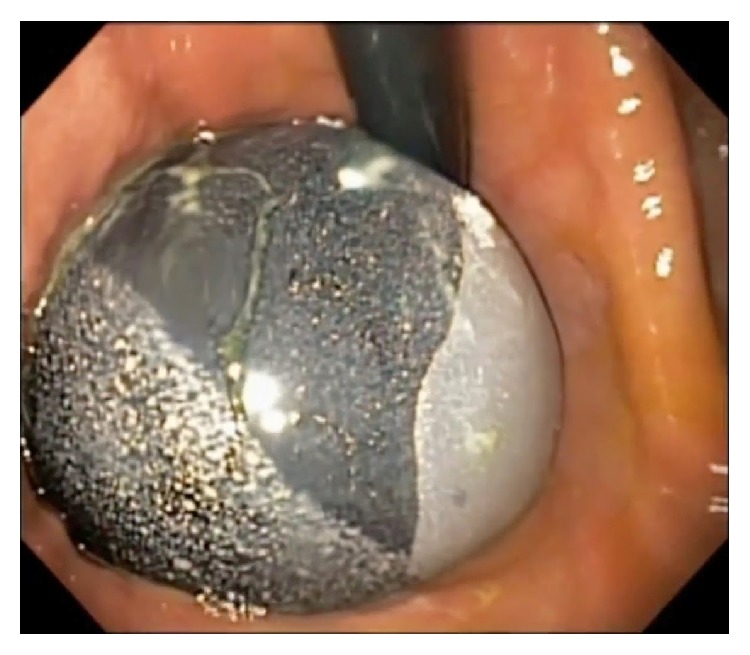
Endoscopic view (in retroflexion) of an intragastric balloon immediately after deployment.

The BioEnterics Intragastric Balloon (BIB, BioEnterics Corporation, USA) became commercially available in 1991 and is the most commonly used balloon for weight loss [27]. It is constructed from a silicone elastomer. The balloon is inserted under endoscopic control into the gastric fundus under light sedation (Figure 1). The balloon is filled with between 400 and 700 mL of normal saline/methylene blue solution via a catheter.

The balloon has been used in those with a body mass index of 40 kg/m^2^ or greater, serving as a pretreatment to bariatric surgery with the aim of reducing anesthetic risk and surgical complications. Other indications include those with lower BMIs with significant comorbidities or in those patients that have contraindications to bariatric surgery [28].

The balloon should be removed after a maximum of six months, beyond this period; the risk of spontaneous balloon deflation significantly increases [29]. The procedure for balloon removal is also performed under sedation. The balloon is punctured with a needle, and the saline is emptied via a catheter. The balloon is removed using grasping forceps or a polypectomy snare.

Several investigators have evaluated the efficacy of the BIB in the management of obesity. In the largest series of BIB patients so far, Genco et al. in a study of 2,515 patients had reported a percentage excess weight loss (%EWL) of 33.8% ± 18.7% at 6 months of follow-up [30]. In this period, there was improvement or resolution of diabetes and hypertension in 86.9 and 93.7% of patients, respectively. The complication rate was acceptable at 2.8% including 5 (0.2%) patients in whom gastric perforation occurred; 2 of whom died.

A recent systematic review of the literature reported on the efficacy of the BIB (now marketed as Orbera (Apollo Endosurgery, Austin, TX)) in obese patients [31]. The review identified 7 studies (409 patients) which reported weight loss at 6 months with a mean EWL of 16 kg. Interestingly, 80% of weight loss was found to occur in the first 3 months of therapy. The durability of this technique is questionable, as demonstrated by Dastis et al. who demonstrated that only a quarter of individuals maintained weight loss up to 30 months after procedure [32].

Two other balloons are now commercially available and these have been specifically designed with antimigration properties. The ReShape Duo (ReShape Medical, San Clemente, CA) consists of two closely attached, independently fluid filled balloons approved for 6-month implantation [33]. The Spatz Adjustable Balloon (Spatz Medical, Great Neck, NY) has an attached catheter to prevent migration and an extractable injection tube for volume adjustment [34]. The adjustable nature theoretically enables the balloon volume to be titrated to tolerability and efficacy. The limited data available on these two balloons show similar efficacy and safety to the Orbera [33, 34].

The most common complications include nausea and vomiting which can persist for up to a week in two-thirds of patients [35]. This can be often effectively managed pharmacologically [35]. Other adverse events include gastric erosion and ulceration. In case of spontaneous balloon deflation, the methylene blue is absorbed and excreted by the kidneys causing green colouration of urine. The location of the balloon must then be verified radiologically and removed endoscopically in order to prevent subsequent small bowel obstruction.

### 2.2. Transpyloric Shuttle

The Transpyloric Shuttle (TPS, BAROnova, Goleta, CA) is a nonsurgical device designed to enable significant weight loss. The TPS is composed of silicone and consists of a large spherical bulb connected to a smaller cylindrical bulb by a flexible catheter. After deployment into the stomach, the TPS moves freely without any physical attachment or invasive anchoring to the tissues. The device is designed to self-position across the pylorus during peristalsis, resulting in intermittent obstruction and resulting in delayed gastric emptying, which induces early and prolonged satiety.

A study of 20 patients by Marinos et al. [36] demonstrated substantial %EWL of 25.1 ± 14.0% and 41.0 ± 21.1% at 3 and 6 months, respectively. Gastric ulceration was a common device related adverse effect, which was noted on scheduled endoscopic evaluation and necessitated device removal in 2 patients. Otherwise, no major gastrointestinal events or complications requiring surgical intervention were seen. Although promising, further studies are required before TPS is widely recommended as a nonsurgical option for weight loss.

## 3. Gastric Restrictive Methods

### 3.1. Transoral Gastroplasty

Transoral gastroplasty is an example of a gastric restrictive procedure, in which suturing or stapling results in gastric partition. Several endoscopic suturing and stapling devices have been developed.

The Endocinch (C.R. Bard, Inc., Murray Hill, NJ) device has been used for endoluminal vertical gastroplasty. This technique involves the use of a suturing device contained within a capsule that is attached to the end of a diagnostic gastroscope. After suctioning tissue into the capsule, a preloaded suture is advanced through the captured tissue. Sutures are deployed in a continuous and cross-linked fashion from the proximal fundus to the distal body of the stomach to create a narrow tube-like passage. Fogel et al. [37] first described the use of this device in 64 obese patients. In that study, 97% of patients achieved >30 %EWL at 12 months of follow-up and the mean %EWL overall was 58% ± 20%. At 12 months, upon repeat endoscopy, only 2 required additional intervention, demonstrating the durability of this technique. There were minimal complications, and patients were discharged on the day of the procedure [37].

An updated version of the device, the RESTORe Suturing System (Bard/Davol, Warwick, RI), has been evaluated in the TRIM trial [38]. This trial was designed in an attempt to validate previously demonstrated degrees of weight loss, with the addition of close clinical and endoscopic follow-up. But, unlike the Endocinch, this device was unable to create a continuous suture pattern owing to suture tension. As a result, this device produced only modest decreases in weight with a mean %EWL of 27.7% ± 21.9% at 12 months of follow-up. Durability was poor with endoscopy at 12 months of follow-up demonstrating partial or complete release of plications in 13 of 14 patients. Modification in suturing techniques or early endoscopic follow-up with repeated interventions may potentially provide a more long-lasting weight loss effect. The procedure was well tolerated without serious adverse events. Of note, neither of the two aforementioned studies included a control group and likely a significant placebo effect related to the procedure and the close monitoring that occurs during a clinical trial influences outcomes.

The TOGA system (Satiety Inc., USA) is a specifically designed device that enables the creation of a stapled, restrictive pouch along the lesser curvature of the stomach under direct endoscopic visualisation. The procedure is performed under general anaesthesia with an average procedure time of 2 hours. The TOGA sleeve stapler is introduced over a guide wire into the proximal stomach. A gastroscope is advanced through the device and retroflexed to directly visualise the stapler. Tissue is gathered into the stapler using suction and staples are delivered. This process can be repeated in order to further narrow the lumen of the sleeve. By slowing the movement of food through the stomach and limiting the ability of the stomach to expand, early satiety is achieved.

Since initial studies conducted by Deviere et al. [39], refinement of technique has resulted in improved outcomes with fewer complications. A prospective, multicenter trial of 67 patients demonstrated 38.1 ± 17.1 %EWL at 12 months with 79% of the study population completing 1-year follow-up. Additionally, improvements in such indices including HbA1c% and triglyceride levels were observed [40]. Most common adverse effects included transient epigastric pain, nausea, and vomiting. More serious complications included one case of respiratory insufficiency and another of pneumoperitoneum, which resolved with conservative management. In 79% of patients who completed one-year follow-up, staple line dehiscence remained a problem, occurring in 50% of patients [40]. Unfortunately, Satiety Inc., the manufacturer of this device, recently declared insolvency and the future of this promising technique is uncertain at this point of time.

**Figure 2:**
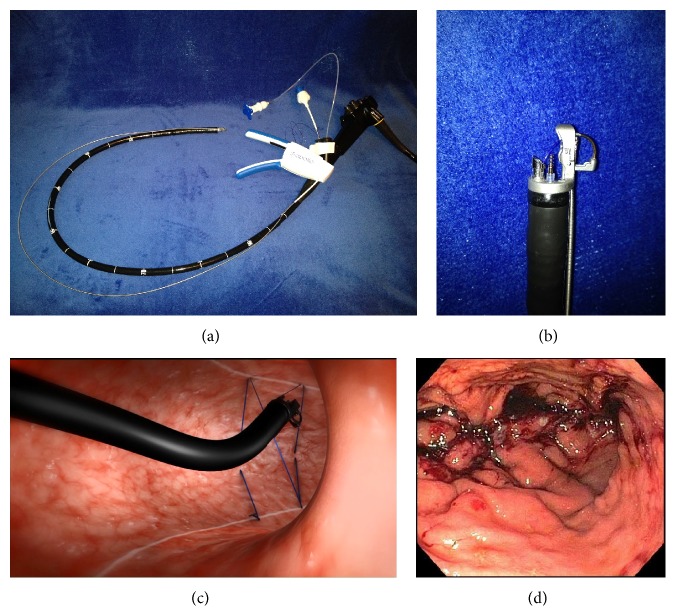
Ex vivo image of the commercially available full thickness suturing system (Overstitch; Apollo Endosurgery, Austin, TX). (a) Image of the device mounted on a double channel gastroscope. (b) A close-up view of the suture mechanism on the tip of the gastroscope. (c) Animation of an efficient suture pattern now performed for endoscopic gastric volume reduction. (d) Endoscopic view of the stomach after the 1st layer of sutures.

In 2013, a study by Abu Dayyeh et al. [41] reported a newer endoscopic suturing device (Overstitch; Apollo Endosurgery, Austin, TX) to perform free hand, full thickness, transoral endoscopic gastric volume reduction in 4 obese patients. In this uncontrolled trial, technical feasibility was demonstrated. The procedure time was over three hours but no intraoperative adverse events were observed. Postprocedural abdominal pain and nausea developed in three patients. Using the same device, a more efficient variation of the suture technique using 8 to 10 sutures has been reported by two groups (Figure 2). A study by Sharaiha et al. performed the same procedure on 10 patients and observed an EWL of 18%, 26%, and 30% after 1, 3, and 6 months, respectively [42]. Another study by Lopez-Nava et al. reported a study in 20 patients and observed an EWL of 29%, 39%, and 54% at 1, 3, and 6 months, respectively [43]. They both concluded that this approach might provide a cost-effective outpatient procedure to add to the steadily growing armamentarium available for treatment of obesity.

Another novel endoscopic stapling technique has recently been examined in its first human phase I study. The new articulating circular endoscopic device (ACE) uses an advanced system with a wide range of motion stapler head that allows physicians to create plications in the desired locations [44]. In this uncontrolled study, treated patients demonstrated a median %EWL of 34.9% over 12 months (IQR 17.8–46.6). One hundred and sixty plications were created in 17 patients without significant complications and endoscopy at 12 months after the procedure suggested that the plications were durable [44]. Long-term follow-up and randomized, controlled studies should evaluate whether this procedure is an effective and durable minimally invasive endoscopic treatment for obesity.

### 3.2. Transoral Endoscopic Restrictive Implant System

The transoral endoscopic restrictive implant system (TERIS) was developed by BaroSense Inc. and introduced as a new endoscopic therapy for the treatment of obesity [45]. The procedure involves placement of a restrictor with a 10 mm central channel for food passage at the gastric cardia, thereby creating a restrictive pouch. The device is left in situ permanently but can be removed or modified if required. Legner et al. [46] conducted a prospective observational study of 13 patients who underwent the procedure. The %EWL was 22% after 3 months. Three patients experienced serious adverse effects with one developing gastric perforation requiring procedural reversal and laparoscopic treatment and 2 others developing pneumoperitoneum. This safety profile remains a concern. Technical improvements and long-term multicenter studies are needed to make TERIS an effective option in the management of obesity.

## 4. Malabsorptive Endoscopic Procedures

### 4.1. Duodenal-Jejunal Bypass Liner (DJBL)

Duodenal-jejunal bypass liner (DJBL) was designed as a nonsurgical approach that enabled components of the Roux-en-Y procedure, namely, exclusion of the duodenum and proximal jejunum and exposure of the distal jejunum to undigested nutrients, reducing absorption and preventing the action of biliary and pancreatic secretions. This action intervenes with the body's metabolic functions, including alteration of incretin pathways resulting in weight loss and improved insulin sensitivity.

The EndoBarrier gastrointestinal liner (GI Dynamics Inc., Lexington, MA, USA) is a flexible, nutrient-impermeable 60 cm sleeve that is anchored in the duodenal bulb and extended into the proximal jejunum deployed using dynamic fluoroscopy. The anchor is a self-expanding stent that enables fixation within the duodenal bulb. The sleeve is maintained from 3 months to 12 months after fixation.

The first reported human case series was by Rodriguez-Grunert et al. [47] in 2008, which reported a 12-week %EWL of 23.6%. Three other studies have completed trials in a randomised fashion against either sham endoscopic procedures or low energy diets. They reported 12-week %EWLs, ranging from 11.9% to 22% with statistically significant weight loss compared with controls [48–50].

Two reports describe longer-term use of the DJBL in obese and diabetic patients. Escalona et al. [51] in a single-arm prospective open-label study reported a mean %EWL of 47% in 24 patients that completed the 52-week program. de Moura et al. [52], in a similarly designed study, used a 52-week HbA1c% as their primary endpoint. This study demonstrated a decrease in HbA1c% of 2.1% ± 0.3%, suggesting a significant effect on diabetes.

These promising results confirmed a recently conducted randomised control trial by Koehestanie et al. [53] in which 70 patients with obesity and type 2 diabetes mellitus were included. Thirty-eight patients were randomised to the DJBL treatment in combination with dietary intervention and 39 controls received dietary intervention alone. At 26 weeks, the DJBL group has a %EWL of 32% (22.0%–46.7%) compared to 16.4% (4.1%–34.6%) in controls (*p* < 0.05). The device was removed at 6 months, and follow-up continued to 52 weeks. At this time, the DJBL group has a %EWL of 19.8% (10.6%–45.0%) compared to 11.7% (1.4%–25.4%) in controls (*p* < 0.05). In addition, the HbA1c% improved to 7.0% (6.4%–7.5%) in the DLBL group compared to 7.9% (6.6%–8.3%) in controls at 6 months (*p* < 0.05).

Importantly, recent systematic reviews have identified a lack of data regarding the durability of the DJBL. There are no studies that examine the effects of the device beyond 52 weeks. The effects of the DJBL on weight loss and diabetes control beyond 52 weeks will need to be investigated further [54, 55].

The device is complicated by a high implantation failure rate of approximately 20% due to anatomical reasons, namely, a short duodenal bulb, and rarely due to investigator inexperience. Complications associated with the procedure most commonly include nausea and upper abdominal pain, which resolved with pharmacological management. More serious complications including device migration and gastrointestinal bleeding require early device extraction. Further studies are needed to analyze the incidence of such complications.

### 4.2. SatiSphere

The endoluminal mechanical device SatiSphere is a new endoscopically implantable device designed to delay transit time of nutrients through the duodenum. It consists of a 1 mm nitinol wire with pigtail ends and several mesh spheres mounted along its course, released into the duodenum and gastric antrum to conform to the duodenal C loop configuration and thereby self-anchor. A randomised controlled trial by Sauer et al. [56] in which the device was inserted in 21 patients demonstrated %EWL of 12.2% at 3 months on intention to treat analysis. This may be as a result of reduced glucose absorption and insulin secretion as well as altered kinetics of GLP-1 levels as demonstrated in a small subgroup of patients who participated in this study. Unfortunately, the study was terminated early due to spontaneous migration of the device occurring in 10 out of 21 patients, two cases requiring surgical intervention. Before widespread use, there is a need for improvement of the device, and an anchoring mechanism may be the solution.

## 5. Regulating Gastric Emptying

### 5.1. Intragastric Botulinum Toxin Injections

**Figure 3:**
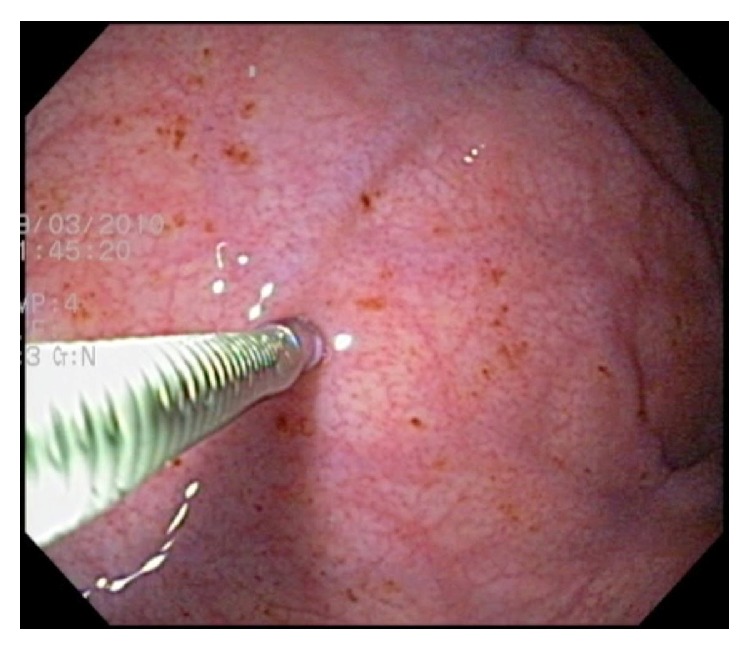
Intragastric botulinum toxin injection. Both the antrum and fundus need to be treated for optimal results.

Botulinum toxin A (BTA) (Figure 3) acts to inhibit acetylcholine release at the neuromuscular junction, hypothetically delaying gastric emptying and inhibiting ghrelin secretion, a potent hormone released from the gastric fundus that stimulates hunger [57]. After successful experiments in rats [58], the first conducted human studies and subsequent randomised controlled trials demonstrated that BTA was safe, but clear benefits for weight loss were not demonstrated, possibly due to variable effects on gastric emptying [59–61].

A slight variation was attempted by Foschi et al. [62] with intraparietal endoscopic administration of BTA into the gastric antrum and fundus (Figure 3). This double-blinded randomised controlled trial demonstrated prolonged gastric emptying and significant weight loss (11 ± 1.09 kg versus 5.7 ± 1.1 kg, *p* < 0.001) after 8 weeks in the BTA group. Most recently, a randomized controlled trial by Li et al. [63] in 20 obese patients demonstrated statistically significant weight loss, ranging from 1 to 12 kg (median of 4.78 kg), and decreased triglyceride levels in those injected with BTA. Gastric emptying times were longer and a decrease in fasting ghrelin levels was appreciated. No complications of the procedure were observed in this study. However, a larger randomised placebo controlled trial by Topazian et al. [64] who enrolled 60 obese patients in a 6-month trial demonstrated a delay in gastric emptying without the effects of early satiety, altered eating behaviours, or weight loss. In this study, only antral injections were performed as opposed to both antral and fundal injections. These results may reflect the relatively short duration of action of BTA and certainly indicate the need for further evaluation of this technique as a method for weight loss.

### 5.2. Gastric Electrical Stimulation

Gastric electrical stimulation has been used in patients with gastroparesis; however, its use in obesity is currently being investigated. Several trials have shown that gastric electrical stimulation results in significant amounts of weight loss due to reduced gastric accommodation, delayed gastric emptying and increased intestinal transit [65–67]. A direct comparison between these studies is difficult, as several different approaches have been attempted including laparoscopic as well as endoscopic placement of electrodes. In addition, some studies have placed electrodes in the stomach and others in the duodenum.

A recent study by Zhang et al. [67] examined the effect of acute retrograde gastric electrical stimulation (RGES) on food intake, gastric accommodation, and gastric emptying in 16 obese patients. They demonstrated that acute RGES reduces caloric intake 759.9 kcal (IQR 547.9–784.9) compared with 985.2 kcal (IQR 842.5, 1063.1) in the sham group. A reduction in gastric accommodation of 16% (*p* = 0.003) was seen in the acute RGES group compared to controls. The difference in gastric emptying rates between the groups did not meet statistical significance. Weight loss was not measured directly in this study, but the study provided meaningful insight into proposed mechanisms of weight loss in patients undergoing gastric electrical stimulation. Although results are encouraging, many questions remain about this modality of therapy and its long-term results.

## 6. Other Techniques

### 6.1. Aspiration Therapy

**Figure 4:**
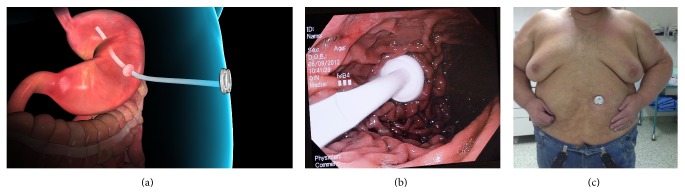
The AspireAssist device used to stimulate weight loss. (a) Animation of the device when placed in situ. (b) Endoscopic view of the AspireAssist device. Note the long, wide-bore intragastric tube to aid in efficient aspiration. (c) The external portion of the device on the skin showing minimal elevation on the skin.

Aspiration therapy is a relatively new technique that involves endoscopic placement of a gastrostomy tube (A-tube) and the AspireAssist siphon assembly (Aspire Bariatrics, King of Prussia, PA) to aspirate gastric contents 20 minutes after meal consumption (Figure 4). A pilot study by Sullivan et al. [68] of 18 obese subjects demonstrated the effectiveness of this technique. After 12 months, subjects in the aspiration therapy group had a %EWL of 49.9% ± 7.7% compared to those with lifestyle therapy alone who achieved a %EWL of 14.9% ± 12.2%. This weight loss was maintained for a further year in 7 of 10 patients who continued with the therapy. The study reported no adverse effects of aspiration therapy on eating behaviour and no evidence of compensation for aspirated calories with increased food intake.

A more recent study by Forssell et al. demonstrated the effectiveness of this device in 25 obese men and women who had the AspireAssist gastrostomy tube placed after 4 weeks of taking a very-low-calorie diet. At 6 months, mean weight loss was 16.5 ± 7.8 kg in the 22 subjects who completed 26 weeks of therapy (*p* = 0.001). The mean %EWL was 40.8 ± 19.8% (*p* = 0.001). No serious complications occurred.

## 7. Conclusion

Obesity and its associated comorbidities are on the rise worldwide, reaching epidemic proportions. To meet this challenge, the field of bariatrics has been growing with the development of minimally invasive surgical procedures. These, however, are subject to considerable cost, limited patient applicability, and substantial risks. Newer bariatric endoluminal interventions have broadened the range of treatment and allow gastroenterologists to play a greater and perhaps central role in the management of obese patients. Preliminary results of these interventions are promising, yet many questions remain regarding the safety and efficacy of such therapies. Additionally, with ongoing innovation, paralleled with clinical research, new treatment options for the endoscopist appear to be on the horizon.

## Abbreviations

BMI: Body mass index
EWL: Estimated weight loss
IGB: Intragastric balloon
DJBL: Duodenal-jejunal bypass liner
BTA: Botulinum toxin A.
